# Extravasation of fluid in arthroscopic shoulder surgery requiring prolonged intubation: a case report

**DOI:** 10.1186/s13037-019-0202-8

**Published:** 2019-06-11

**Authors:** Brandon R. Vier, Kyle W. Mombell, Erin L. Gagliano, Nicole M. King, Lucas S. McDonald

**Affiliations:** 1 Department of Orthopedic Surgery, Naval Medical Center San Diego, 34800 Bob Wilson Dr, San Diego, CA USA; 2 Department of Anesthesia, Naval Medical Center San Diego, 34800 Bob Wilson Dr, San Diego, CA USA

**Keywords:** Shoulder, Arthroscopy, Fluid extravasation, Airway

## Abstract

**Background:**

Shoulder arthroscopy is a safe and effective procedure with a low complication rate. Although rare, there are potentially life-threatening risks such as fluid extravasation causing airway compromise.

**Case presentation:**

We report the case of a 65-year-old female treated with an arthroscopic rotator cuff repair who had significant extravasation of arthroscopic fluid causing severe facial and neck swelling. Overnight intubation was required for respiratory monitoring until the edema had resolved enough to allow safe extubation.

**Conclusion:**

This case highlights the risk factors and clinical course of a patient with airway compromise caused by extravasation of fluid during shoulder arthroscopy. Although shoulder arthroscopy is a safe procedure, surgeon familiarity with the risk factors for this complication and close monitoring can aid in its identification and allow for appropriate treatment.

## Background

Shoulder arthroscopy facilitates a minimally invasive approach to the joint compared to open procedures which are inherently more traumatic to the soft tissues. Arthroscopy has its own unique complications primarily related to the need for distension of the operative joint with pressurized irrigation fluid to aid in visualization. Extravasation of irrigation fluid and even air into the surrounding soft tissues has previously been with the most significant consequence of this extravasation is edema resulting in airway compromise [1].

Most of the earlier published cases involved patients undergoing arthroscopy under regional anesthesia where the patients vocalized symptoms of impending airway compromise [2–6], but more recent literature describes cases with general anesthesia where the edema isn’t recognized until just prior to or even after extubation/removal of an laryngeal mask airway (LMA) [7–9]. Edema can be quite profound and extend into the chest, neck, and even into the face making the tissue tense and cool to palpation. In the majority of cases, the patient remains intubated, or if needed, is urgently intubated for airway protection and monitored for 24 to 48 h until the edema has sufficiently resolved to permit safe extubation.

While the glenohumeral joint has a capsule that may provide an anatomic barrier to fluid extravasation, it is has been postulated that the lack of such a capsule in the subacromial space allows for fluid extravasation during arthroscopy [10, 11]. Anatomical variants also exist including aberrant connections between the glenohumeral joint and soft tissues of the neck via the infraspinatous fossa [7]. Additionally, rotator cuff tears and iatrogenic anterior deltoid tears are potential risk factors for fluid extravasation [4].

A multitude of other risk factors are identified in the literature. Obese patients have multiple tissue planes that may allow fluid to spread more easily into the surrounding soft tissue [4]. Patient positioning in the lateral decubitus position increases the risk of significant edema in the neck due to its dependent position during the procedure [2]. High pump pressures and prolonged surgical times have are additional risk factors for fluid extravasation [2, 6, 8, 10]. Deltoid pressure monitoring during arthroscopy demonstrated that the intramuscular pressure significantly increases during arthroscopic acromioplasty, routinely reaching 50–120 mmHg, and that adduction and external rotation of the shoulder cause these pressures to elevate up to 150 mmHg^10^.

We describe a case where arthroscopic rotator cuff repair in a patient with several risk factors for fluid extravasation resulted in facial and neck edema requiring prolonged intubation and respiratory monitoring overnight.

The patient provided written consent for publication.

## Case presentation

**Fig. 1:**
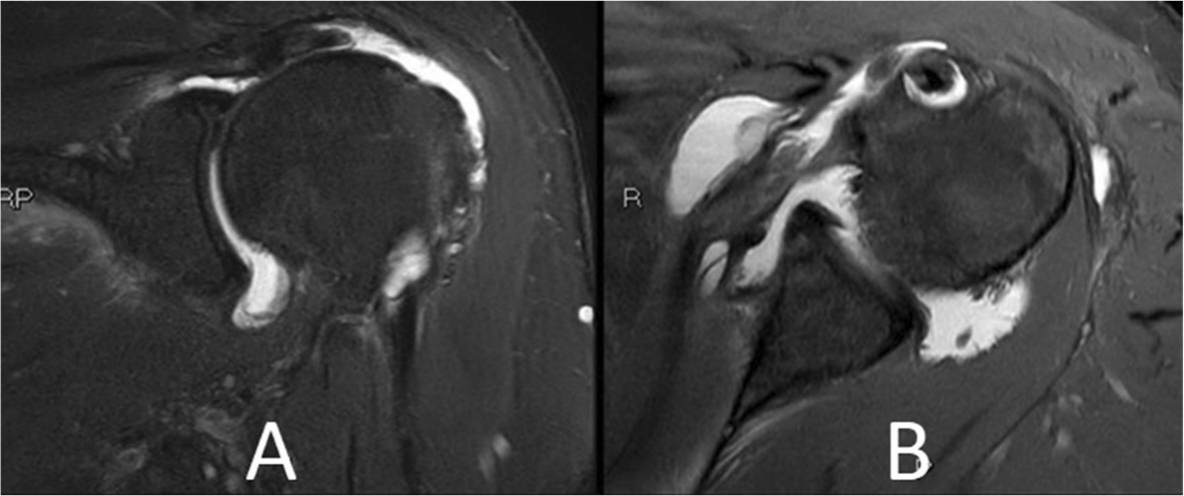
Magnetic Resonance Imaging (MRI) images depicting the retracted full-thickness tear of the supraspinatus tendon (**a**) and partial-thickness tear of the upper border of the subscapularis tendon (**b**)

A 65-year-old female with a body mass index of 29 presented with a 7-month history of left shoulder pain and weakness. Physical exam and diagnostic imaging were consistent with a symptomatic full thickness rotator cuff tear of the supraspinatus and a partial tear of the subscapularis tendon (Fig. 1). She was indicated for an elective arthroscopic surgical repair. She was otherwise healthy with her only medical comorbidity consisting of hyperlipidemia. She had no prior surgical history including no prior shoulder procedures.

**Fig. 2:**
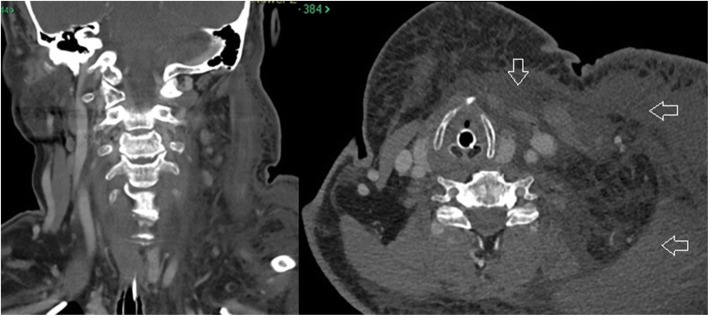
CT scan showing paratracheal edema

Anesthesia evaluation on the day of surgery was performed and her neck was recorded as “unremarkable.” She was assigned an American Society of Anesthesiologists (ASA) score of 2. On the day of surgery, she was easily intubated with an endotracheal tube in the supine position and then placed in the standard lateral decubitus position for arthroscopic shoulder surgery. Exam under anesthesia was performed followed by a diagnostic shoulder arthroscopy. Normal saline was fed into a Stryker CrossFlow® Integrated Arthroscopy Pump (Stryker Endoscopy, San Jose, CA, USA) set at 25 mmHg initially. Epinephrine was not added to the irrigation fluid. Shortly after beginning the case, the arthroscopic fluid pressure was raised to 35 mmHg to aid in visualization where it remained for the duration of the case. No lavage cycles were utilized. Standard posterior, anterosuperior and anteroinferior portals were placed as well as a lateral working portal. She was found to have a type 1 superior labrum anterior to posterior (SLAP) tear, degenerative changes in the anterior, inferior and posterior labrum, a subscapularis tear in the upper one third which was retracted medially, and a complete supraspinatus tear. A biceps tenotomy was performed followed by rotator cuff repair of the subscapularis and supraspinatus tears utilizing suture anchors. Bone quality was remarkably poor with pull-out of multiple suture anchors during the rotator cuff repair adding to surgical complexity and time. Total operative time was 3 h and 53 min. Upon completion of the case and removal of the surgical drapes, significant unilateral face and neck swelling was noted on the side of the operative shoulder (the non-gravity dependent side). Upon consultation with the anesthesia providers the decision was made to obtain a computed tomography (CT) scan for visualization of the soft tissues surrounding the airway, with a plan to leave the patient intubated overnight. The CT demonstrated diffuse soft tissue edema in the subcutaneous tissues of the neck, chest and face. The airway was deviated at the level of the trachea due to the paratracheal edema. There was no focal collection or extravasated contrast indicative of a hematoma or vascular injury (Fig. 2).

**Fig. 3:**
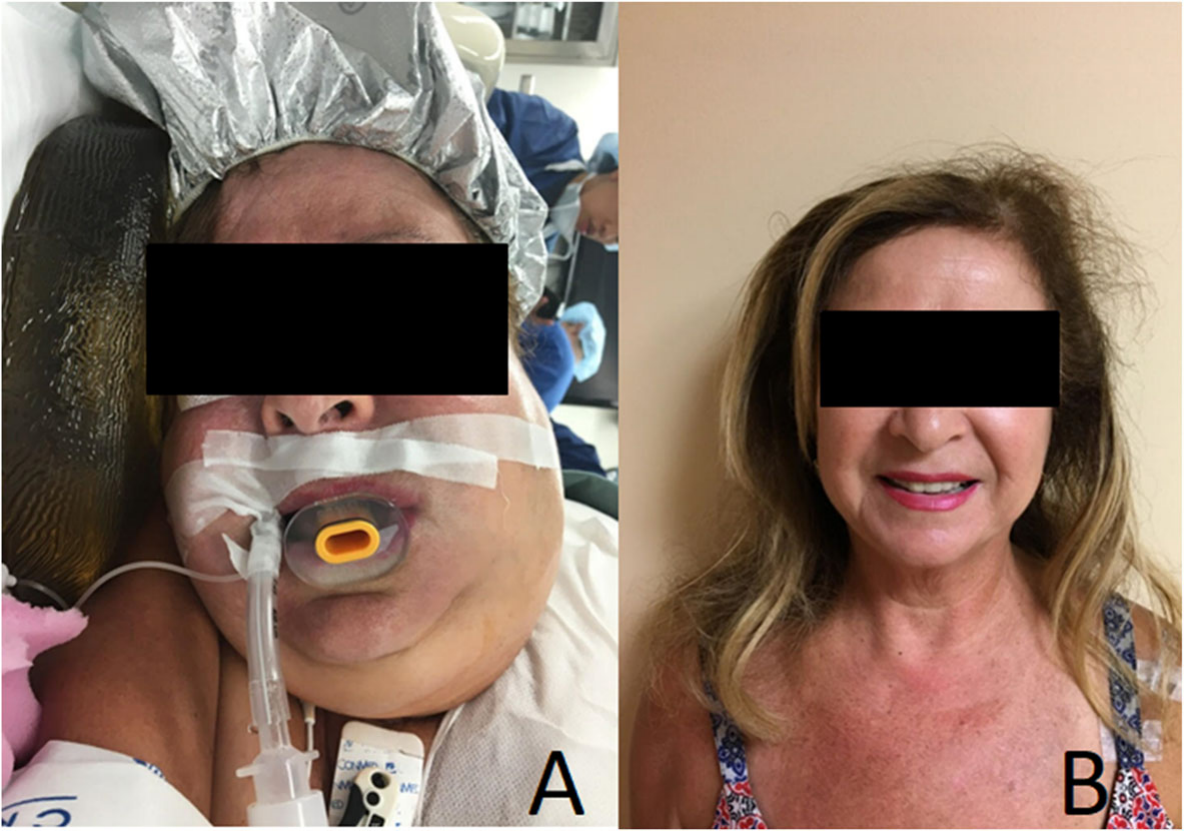
Clinical photo showing intraoperative facial edema (**a**) and complete resolution at two week follow up (**b**)

The patient was monitored overnight in the Intensive Care Unit (ICU). After resolution of the edema, she met standard ICU extubation criteria, and was extubated on the morning of postoperative day one. Postoperatively, the patient has done well without any airway or pulmonary complications, complete resolution of preoperative symptoms, and return to baseline shoulder function (Fig. 3).

## Discussion

In this case, the patient was intubated for the duration of the procedure, which ensured airway protection and obviated the need for intubation upon recognition of the condition. Had the procedure been performed under regional anesthesia with sedation or with a laryngeal mask airway (LMA) a conversion to an endotracheal airway would have been required. Fortunately, inspection of the neck and surrounding soft tissues prior to extubation identified this complication and the patient remained intubated in a monitored setting until extubation was safe.

A CT of the neck and chest was requested by the anesthesia team to assess for laryngeal edema. Additional strategies exist to assess for a potentially compromised airway. If the edema is noted before extubation of the patient, a cuff-leak test can be performed. This involves deflating the cuff of the endotracheal tube and then digitally occluding it. If the patient is able to breath around the deflated cuff, it is hypothesized they are at lower risk for post-extubation stridor and need for reintubation. Results of this test have varied widely depending on patient population and study so results of the test should be utilized in conjunction with other clinical tools [12, 13]. Furthermore, if the edema is noted during the case, the patient can be repositioned to the beach chair position to allow gravity to pull the fluid away from the airway, or the case can be converted to an open procedure.

Established risk factors for fluid extravasation present in this case included lateral decubitus positioning, prolonged operative time, and performing a subacromial decompression. These represent modifiable risk factors and are important preoperative and intraoperative considerations for the treating surgeon. Adequate visualization of the surgical field during shoulder arthroscopy is a necessity, but the surgeon should have an understanding of the risks of elevated pump pressures and liberal use of lavage cycles. Visualization of the subacromial space can be aided by narrowing the pressure differential between the arthroscopic pressure in this space and the systolic blood pressure. Morrison et al. demonstrated that the visual clarity of the subacromial space in the lateral decubitus position was excellent as long as there was a pressure difference of less than 49 mmHg. This pressure differential can be maintained through a combination relative hypotensive anesthesia and higher pump pressures [14, 15]. The surgeon and anesthesia provider must make a conscious effort to balance these two variables in order to maintain the visualization necessary for surgery but avoid complications from excessive hypotension or extreme pump pressures.

Although the risk of fluid extravasation may be decreased with the beach chair position, this positioning is also not without risk. Beach chair positioning carries a higher risk of cerebral hypoperfusion especially with relative hypotensive anesthesia. It also carries higher rates of venous and arterial air embolism, and even quadriplegia from incorrect head positioning during surgery [14, 15].

Memon et al. conducted a systematic review of the published literature concerning fluid extravasation as result of shoulder arthroscopy. Approximately 65% of the patients who experienced fluid extravasation were positioned in the lateral decubitus position compared to 30% in the beach chair position. The authors also suggest limiting the pump pressures to less than 150 mmHg. Utilizing a gravity feed irrigation setup may be a safer alternative to pump systems which result in higher intra-articular pressures [16–18]. Their literature review also found that symptomatic patients had irrigation volumes of between 20 and 36 L and suggest limiting the total irrigation volume to 20 L. [16]

Total operative time is directly proportional to the volume of irrigation fluid used and subsequent weight gain by the patient, presumably from absorption of the irrigation fluid [19]. Although there is no clear safe upper limit for operative times, some authors suggest limiting surgical time to between 90 and 120 min to reduce this risk [16, 20] .

Fluid extravasation not only affects the local soft tissues but may have other systemic complications such as electrolyte disturbances. Ichai et al. demonstrated that there is systemic absorption of glycine irrigation fluid during shoulder arthroscsopy. Although the absorption of glycine may be different than that of other irrigation fluids including normal saline or Ringer’s lactate, the authors suggest that the risk of significant systemic absorption can be mitigated with lower pump pressures and a lower volume of irrigation fluid used during arthroscopy [16, 21].

## Conclusions

Shoulder arthroscopy is a generally safe and effective procedure, but surgeons and anesthesia providers must be aware of the rare and potentially life-threatening complication of fluid extravasation and airway compromise. Risk factors such as lateral decubitus positioning, obesity, high pump pressures, and long operative times may increase this risk. Surgeons and anesthesia providers should consider routine endotracheal intubation in arthroscopic shoulder procedures with multiple risk factors for dangerous fluid extravasation.

## Data Availability

Not applicable.

## Abbreviations

ASA: American Society of Anesthesiologists
CT: Computed Tomography
ICU: Intensive Care Unit
LMA: Laryngeal Mask Airway
MRI: Magnetic Resonance Imaging
SLAP: Superior Labrum Anterior to Posterior
